# The Axonal Cytoskeleton and the Assembly of Nodes of Ranvier

**DOI:** 10.1177/1073858417710897

**Published:** 2017-05-23

**Authors:** Aniket Ghosh, Diane L. Sherman, Peter J. Brophy

**Affiliations:** 1 Centre for Neuroregeneration, University of Edinburgh, Edinburgh, UK

**Keywords:** node of Ranvier, myelin, neurofascin, oligodendrocyte, heminode, axonal cytoskeleton

## Abstract

Vertebrate nervous systems rely on rapid nerve impulse transmission to support their complex functions. Fast conduction depends on ensheathment of nerve axons by myelin-forming glia and the clustering of high concentrations of voltage-gated sodium channels (Nav) in the axonal gaps between myelinated segments. These gaps are the nodes of Ranvier. Depolarization of the axonal membrane initiates the action potential responsible for impulse transmission, and the Nav help ensure that this is restricted to nodes. In the central nervous system, the formation of nodes and the clustering of Nav in nodal complexes is achieved when oligodendrocytes extend their processes and ultimately ensheath axons with myelin. However, the mechanistic relationship between myelination and the formation of nodal complexes is unclear. Here we review recent work in the central nervous system that shows that axons, by assembling distinct cytoskeletal interfaces, are not only active participants in oligodendrocyte process migration but are also significant contributors to the mechanisms by which myelination causes Nav clustering. We also discuss how the segregation of membrane protein complexes through their interaction with distinct cytoskeletal complexes may play a wider role in establishing surface domains in axons.

## Introduction

Complex nervous systems require rapid nerve impulse transmission. Myelinated nerves in the vertebrate nervous system display two closely related features that together promote fast conduction: the first is the ensheathment of their axons by specialized glia to form the multilamellar myelin sheath, and the second is the clustering of axonal voltage-gated sodium channels (Nav) at the nodes of Ranvier in response to myelination. These two key evolutionary adaptations promote rapid saltatory conduction as first described in 1949 (Huxley and Stämpfli 1949). Hence, axon-glia interaction leading to axon ensheathment has a critical role in vertebrate neural development. Here we focus on the central nervous system (CNS) where oligodendrocytes ensheath axons because recent work has shed new light on how these myelin-forming glia participate in clustering Nav at the CNS node.

## The Nodal Complex and the Paranodal Axoglial Junction

**Figure 1.**
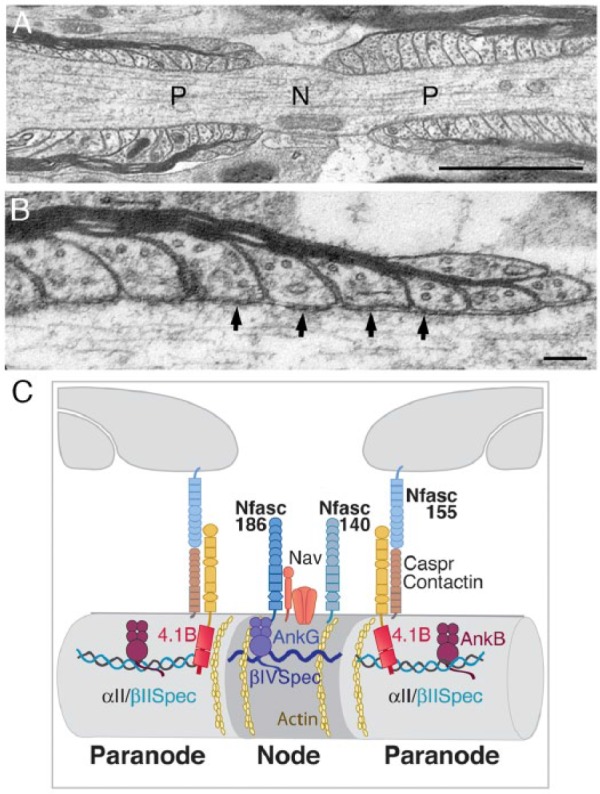
(A) Electron micrograph of a myelinated axon from the ventral funiculus of the spinal cord in longitudinal section shows the node (N) and paranodal (P) domains. Bar = 5 µm. (B) Paranodal loops between the myelin sheath and the axolemma at high magnification display septate junctions of the CNS axoglial complex (arrows). Bar = 1 µm. (C) The major proteins of the CNS node and paranodal domains interact with distinct components of the axonal cytoskeleton.

During myelination the pore-forming sodium channel α subunits and their associated proteins become clustered at the nodes of Ranvier. In addition to the α and accessory β subunits of the channel the CNS nodal complex includes two neuronal isoforms of Neurofascin, Nfasc186 and Nfasc140, Contactin, potassium channels, and two proteins that are believed to link the complex with the underlying actin cytoskeleton, βIV spectrin and AnkyrinG (Bekku and Oohashi 2010; Berghs and others 2000; Davis and others 1996; Jenkins and Bennett 2002; Kazarinova-Noyes and others 2001; Waxman and Ritchie 1993; Zhang and others 2015) (Fig. 1). A complex extracellular matrix probably of glial origin, and predominantly comprising proteoglycans, surrounds the CNS node and participates in node stabilization (Rasband and Peles 2015).

Nodes of Ranvier are flanked by junctional adhesion complexes between the lateral extremities of the myelin sheath and the axon itself (Fig. 1). They are the largest intercellular adhesions found in vertebrates and are the primary zones of interaction between the ensheathing glial cell and the axolemma. Paranodal axoglial adhesions are characterized by the presence of septate junctions that are clearly visible in electron micrographs (Fig. 1). These septate structures reveal an extracellular adhesion complex that is composed of three membrane proteins, the glial isoform of Neurofascin, Nfasc155, and the axonal proteins Caspr (also known as Paranodin) and Contactin (Bhat and others 2001; Boyle and others 2001; Charles and others 2002; Collinson and others 1998; Einheber and others 1997; Menegoz and others 1997; Sherman and others 2005; Tait and others 2000). Glial Nfasc155 and the two neuronal Neurofascin isoforms located at the node, Nfasc186 and Nfasc140, are all encoded by the *Neurofascin* (*Nfasc*) gene.

## Nodal and Paranodal Proteins Essential for CNS Node Assembly

Although several other proteins participate (Rasband and Peles 2015), the proteins encoded by the *Nfasc* gene are uniquely essential for the clustering of Nav at nodes of Ranvier (Sherman and others 2005). In the absence of an intact paranodal axoglial junction, neuronal Neurofascins can cluster Nav at the node (Amor and others 2017; Sherman and others 2005; Zhang and others 2015; Zonta and others 2008). Furthermore, and perhaps surprisingly, the paranodal axoglial junctional complex of Nfasc155, Caspr, and Contactin can also cluster Nav at nodes independently of neuronal Neurofascins (Amor and others 2017; Zonta and others 2008). This presumably reflects a degree of redundancy to ensure that Nav become appropriately concentrated at nodes during myelination. The importance of Nav clustering at the node is shown dramatically in mice lacking a functional *Nfasc* gene (Sherman and others 2005). Without neuronal Neurofascins and an intact axoglial junction due to the absence of Nfasc155 nerves undergoing myelination are unable to transition to saltatory conduction and the mice die at postnatal day 7.

How the nodal Neurofascins cluster Nav at CNS nodes is still unclear. In contrast, significant progress has been made in identifying the mechanisms by which the paranodal axoglial junctional complexes, together with their underlying axonal cytoskeleton, participate in node formation.

## The Paranodal Cytoskeleton

Oligodendrocytes extend processes that then migrate along the surface of the axons they will ensheath. Mature nodes are formed when contiguous glial processes converge. The axoglial complex, comprising glial Nfasc155 and the axonal proteins Caspr and Contactin, assembles at an early stage at the tips of migrating processes and promotes the convergence of glial processes (Brivio and others 2017; Zonta and others 2008) (Fig. 1). Recently, it has become clear that the axon, and in particular the paranodal axon cytoskeleton, also has a role in oligodendrocyte process extension and therefore in CNS node formation (Brivio and others 2017).

In mature myelinated nerves, there is a distinct paranodal axonal cytoskeleton that includes the actin-binding proteins αII spectrin, βII spectrin, AnkyrinB, and Protein 4.1B (Ogawa and others 2006) (Fig. 1). This intra-axonal cytoskeletal complex appears to influence domain organization at the axolemmal surface since loss of paranodal βII spectrin leads to mislocalization of adjacent juxtaparanodal proteins, such as potassium channels, even when the extracellular axoglial junction is intact (Zhang and others 2013). This suggests that the boundaries between cytoskeletal complexes within the axon can have profound effects on protein disposition at the axonal surface.

## The Interface between the Nodal and Paranodal Axonal Cytoskeleton: An Organizing Principle

Deletion of axonal membrane proteins known to interact with cytoskeletal-associated proteins has led to the idea that the association of mature nodal and paranodal domains is mediated via the axonal cytoskeleton. Neuronal Nfasc186 interacts with AnkryinG (Davis and others 1996) and can be selectively reduced from CNS nodes by approximately 95% by inducible conditional Cre-mediated inactivation of a floxed allele of the *Nfasc* gene in *ThyCreERT2/Nfasc^fl/−^* mice (Desmazieres and others 2014). Loss of neuronal Neurofascin in the CNS is accompanied by a 70% to 80% loss in AnkyrinG and βIV spectrin. Importantly, this substantial loss of nodal cytoskeleton proteins is associated with severe disruption of the adjacent paranodal axoglial complex. Paranodal Caspr is lost and Kv channels that are normally restricted to the juxtaparanode, a zone more distal to the node, invade the paranodal domain. This disruption of adjacent domains points to an intraxonal cytoskeletal architecture with a role in stabilizing cell surface domains.

Paranodal junctions in the peripheral nervous system (PNS) are much less susceptible to disruption after almost complete loss of nodal Nfasc186 (Desmazieres and others 2014). However, this may reflect the fact that the accompanying loss of nodal AnkyrinG and βIV spectrin is much less pronounced (40% to 50%) than in the CNS. Hence, one might expect that the cytoskeletal interface at the node-paranode border would be much less disrupted in the axon in the PNS compared to the CNS. More recent studies have confirmed that although loss of both Nfasc186 and βII spectrin depletes AnkyrinG and βIV spectrin at the PNS node, the extracellular paranodal junctional complex appears to remain intact (Amor and others 2017). This supports the view that the adhesive complex between Schwann cells and axons at the paranode may be more resistant to disruption than its CNS counterpart.

## Efficient Oligodendrocyte Process Extension Requires Linkage of the Axoglial Complex to the Axonal Cytoskeleton

The concentration of the axoglial junctional complex at the leading tips of oligodendrocyte processes in contact with the axon suggests that one or more of the components of the complex might act as anchorage points for dynamic cytoskeletal elements, either glial, or axonal, that drive the movement of myelinating processes (Zonta and others 2008).

The cytoplasmic tail of Nfasc155 has a binding site for the actin-binding protein AnkyrinG (Davis and others 1996). Nevertheless, although AnkyrinG is present in oligodendrocytes at paranodes (Chang and others 2014), glial Nfasc155 lacking a C-terminus promotes normal oligodendrocyte process extension (Zonta and others 2008). Therefore, we can conclude that a direct link between the junctional protein Nfasc155 and the oligodendrocyte cytoskeleton is unlikely to drive process migration, at least not via AnkyrinG.

On the axonal side of the junction Contactin is a GPI-linked protein and cannot be directly linked to the axonal cytoskeleton. However, the cytoplasmic tail of Caspr has a Protein 4.1B binding site and is therefore a candidate for linking the axoglial complex to the axonal cytoskeleton (Buttermore and others 2011; Denisenko-Nehrbass and others 2003; Einheber and others 1997; Horresh and others 2010; Menegoz and others 1997; Ohara and others 2000). Like other members of the Protein 4.1 family, Protein 4.1B has a spectrin/actin binding domain and is present in a complex with αII and βII spectrin tetramers at the paranode in the CNS (Garcia-Fresco and others 2006; Ogawa and others 2006). Consequently, Caspr, through its interaction with the αII and βII spectrin/actin cytoskeleton via Protein 4.1B, is a strong candidate for participating in the anchoring of the axoglial junctional complex to the propulsive power of the axonal cytoskeleton during oligodendrocyte process extension.

Recent studies have indeed shown that the interaction between Caspr and Protein 4.1B does have a function in oligodendrocyte process extension. When the C-terminus of Caspr is missing or axons lack Protein 4.1B there is a substantial delay in process migration (Brivio and others 2017). Importantly, preventing the interaction of Caspr with Protein 4.1B has no effect on the ability of the extracellular domain of this mutant ΔC-Caspr to reconstitute the axoglial complex and reform septate junctions in Caspr-null mice (Brivio and others 2017). Hence, the delay in oligodendrocyte process extension observed when the axoglial junction is disrupted in the absence of Nfasc155 is most likely due to disruption of the axonal cytoskeleton that normally underlies the tip of the oligodendrocyte process (Zonta and others 2008). Hence, we can conclude that linkage of the oligodendrocyte processes to the underlying axonal cytoskeleton influences process extension. However, does it also affect the assembly of the nodal complex itself?

## Mechanisms of CNS Heminode Formation and the Underlying Axonal Cytoskeleton

**Figure 2.**
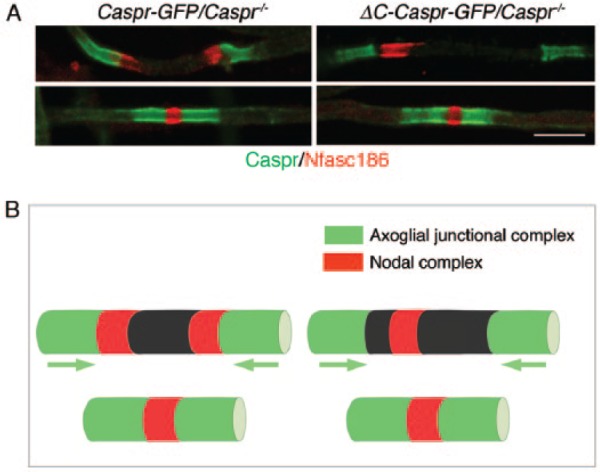
(A) Immunofluorescence showing that nodal proteins are mislocalized during oligodendrocyte process migration when Caspr lacks its cytoplasmic C-terminus (Caspr mutant) and is therefore unable to interact with Protein 4.1B. Both full length Caspr fused to GFP at its C-terminus (Caspr-GFP) and C-terminally truncated Caspr ΔC-Caspr were expressed in transgenic mice lacking a functional Caspr gene (*Caspr^−/−^)*. Caspr at the paranodes is in green, the nodal complex visualized with antibodies to Nfasc186 is red. Mutant Caspr is unable to assemble symmetrical heminodal complexes as seen for full length Caspr. However, mature nodes are eventually formed. Bar = 5 µm. (B) Diagrammatic representation of the location of nodal and axoglial complexes during oligodendrocyte process migration. As seen on the right, decoupling Caspr from its interaction with Protein 4.1B releases the nodal complex from its association with axoglial complexes at the leading edge of each oligodendrocyte process. Although migration of the processes is highly retarded they ultimately form a node of Ranvier.

During peripheral nerve myelination nodal proteins are first detected adjacent to the extremities of growing Schwann cells; these clusters are referred to as heminodes and their assembly is believed to increase the efficiency of mature node formation (Schafer and others 2006). Symmetrical heminodal complexes are also observed at the tips of migrating oligodendrocyte processes in close contact with the axoglial complex (Brivio and others 2017). However, when the axoglial adhesion complex is uncoupled from Protein 4.1B or Protein 4.1B is absent symmetrical heminodes are no longer formed in the CNS; instead, a single nodal complex is found between the converging processes (Brivio and others 2017) (Fig. 2).

Why should dissociating the axoglial complex from its associated axonal cytoskeleton at the tips of oligodendrocyte processes influence the localization of the nascent clusters of nodal proteins? One possibility is that the distinctive actin-associated cytoskeleton comprising Protein 4.1B/αII/βII spectrin underneath the axoglial complex forms a functional interface with actin-associated βIV spectrin and AnkyrinG underlying the nodal complex. This interface would not only function as a boundary, but would also serve as a dynamic interface ensuring that nascent nodal complexes were anchored adjacent to the tips of myelinating processes.

**Figure 3.**
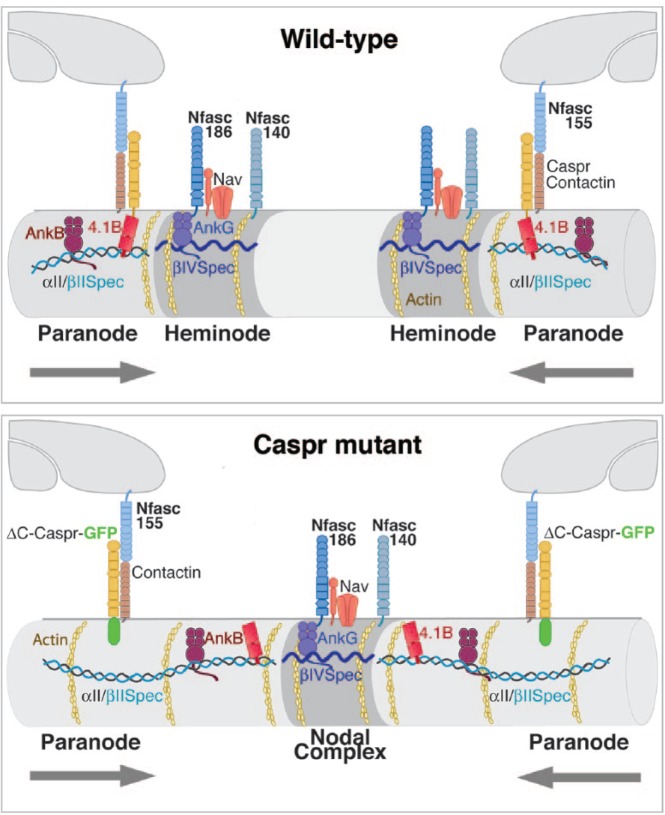
The nodal complex and axoglial complexes in wild-type CNS axons are associated with distinct submembranous cytoskeletal complexes. The interaction of the axoglial complex with αII and βII spectrin and their associated actin is normally anchored through Protein 4.1B. This establishes a cytoskeletal interface with βIV spectrin and its associated actin, possibly through the competition of these different spectrin types for actin. When Caspr lacks its cytoplasmic C-terminus (Caspr mutant) the Protein 4.1B-αII spectrin-βII spectrin complex is no longer anchored at the axoglial complex. However, the interface with the cytoskeleton associated with the nodal complex persists and results in a single nodal complex no longer tethered to each myelinating process rather than two heminodes.

If this dynamic interface concept reflects reality then a prediction can be made. When the “axoglial cytoskeleton” is no longer anchored at the extracellular axoglial adhesion junction, either through uncoupling Caspr from Protein 4.1B or through loss of Protein 4.1B, Protein 4.1B, and βII spectrin should no longer be restrained there and would now invade the axon between converging myelinating oligodendrocyte processes. Nevertheless, their cytoskeletal proteins should still form an interfacial boundary with clustered nodal proteins. Since invasion of Protein 4.1B and βII spectrin would occur on both sides of the gap between migrating processes, the consequence would be a single nodal complex from which Protein 4.1B and βII spectrin remain excluded. This is exactly what is observed (Brivio and others 2017) (Figs. 2 and 3).

## Implications of the “Dynamic Cytoskeletal Interface” for the Establishment of Other Axonal Membrane Domains

The axon initial segment (AIS), the proximal domain of the axon, is enriched in proteins also found at nodes of Ranvier including Nav, Nfasc186, βIV spectrin, and AnkyrinG. It has been argued that αII/βII spectrin and their associated axonal cytoskeletal proteins contribute to an intra-axonal boundary that excludes βIV spectrin- and AnkyrinG-associated proteins such as Nfasc186, and that this defines the extent of the AIS (Galiano and others 2012).

Recent studies have strongly suggested that such a boundary at the first heminode at the distal extremity of the AIS does indeed exist. Super-resolution microscopy shows that the actin cytoskeleton and βIV-spectrin are periodically clustered with a periodicity of 190-nm along the AIS. Spectrin tetramers show a repetitive patterning of ~180 to 190 nm and are aligned longitudinally along the axon to connect adjacent ring-like actin structures (D’Este and others 2015; Leterrier and others 2015). Furthermore, Nav and Nfasc186 are distributed in a manner that is coordinated with the underlying actin-spectrin cytoskeleton in the AIS (D’Este and others 2015). This periodic patterning restricts membrane protein motion in the AIS and mathematical modeling further suggests that there is diminished lateral mobility due to a diffusion barrier between these segments (Albrecht and others 2016). By analogy with what we now know about the CNS axoglial junction in node formation, it will be of great interest to determine how the αII /βII spectrin tetramers are anchored at the first heminode of the distal region of the AIS and how they contribute to that interface.

A fundamental question remains. How are the distinct axonal cytoskeletal complexes that associate with nodal proteins and the axoglial complex segregated. Furthermore, how do these distinct cytoskeletal complexes remain in close apposition even when the axoglial cytoskeletal complex is uncoupled from its overlying axonal membrane domain? Any possible mechanism must account for the fact that even when the Protein 4.1B/αII /βII spectrin complex is no longer tethered to the axoglial complex it remains closely apposed to the nodal region while still largely excluded from the nodal cytoskeleton (Brivio and others 2017).

In PNS axons super-resolution microscopy has confirmed that the βIV spectrin and βII spectrin actin cytoskeletons abut at the node (D’Este and others 2017). Nevertheless, in Protein 4.1B-null PNS axons βII spectrin can mix with the βIV spectrin/actin cytoskeleton at the node (Cifuentes-Diaz and others 2011). This suggests that Protein 4.1B may restrict the propensity of the distinct spectrin/actin complexes to mix, thus setting up the cytoskeletal interface. Uncoupling the Caspr-Protein 4.1B-βII spectrin complex from the axoglial junction may change the periodic patterning of βII spectrin. This could then result in a change in its diffusion barrier properties and thus promote invasion into the axon between converging myelinating oligodendrocyte processes. Whether a similar periodic lattice of spectrins exists at CNS nodes and paranodes and whether they account for similar barrier properties as found in the AIS awaits future experimentation.
